# Are Universities Ready to Support Autistic Students? A Call to Increase Coordinated Campus Efforts

**DOI:** 10.1089/aut.2024.0293

**Published:** 2025-08-11

**Authors:** Meghan G. Blaskowitz, Alia M. Pustorino-Clevenger, McKenna Killion, Cassidy Shirlow

**Affiliations:** ^1^ Compass Inclusive Education Program, Duquesne University, Pittsburgh, Pennsylvania, USA.; ^2^ Millersville University, Millersville, Pennsylvania, USA.; ^3^ TEIS Early Intervention Services, Pittsburgh, Pennsylvania, USA.

**Keywords:** autistic students, autism, autism spectrum disorder (ASD) diagnosis, postsecondary education, inclusive education, Universal Design for Learning (UDL), accommodations, best-practice supports

## Abstract

Over the past decade, universities have seen an increase in autistic student enrollment; however, many of these universities are not supporting autistic students to have a quality college experience. Although more autistic young adults are pursuing college, many do not persist to graduation due to a lack of individualized accommodations, siloed provision of support from universities, and overall hesitancy among faculty, staff, and administration to include autistic students in all campus spaces. In addition, autistic students struggle to build self-advocacy, self-determination, and functional life skills upon transition from high school. This *Perspectives* article was written by a team of inclusive postsecondary education professionals who have seen firsthand the challenges that autistic students experience in college. These authors advocate for increased coordination and collaboration among university units to promote greater inclusion and targeted support for autistic students on their campuses. These recommendations include employing a student-centered approach to understanding and supporting autistic students, training faculty on autism and Universal Design Learning practices, and intentionally embedding opportunities for autistic students to enhance their social–emotional learning and on-campus relationships. Our direct experiences in trialing these systems of support have cultivated more accepting and positive environments for autistic students. Neurodivergent students deserve the same access and opportunity to seek higher education as their neurotypical peers. This article presents actionable steps for how this can happen on college campuses.

## Abstract (plain language summary)

Community Brief

Why is this topic important?

More autistic young adults are pursuing postsecondary education at colleges and universities across the U.S. than ever before. It is imperative that campuses, faculty, and staff equip themselves with proper supports and resources to offer neurodivergent students a quality postsecondary experience.

What is the purpose of this article?

This article recommends coordinated university approaches across academic units to give autistic students greater opportunities to succeed in college.

What personal or professional perspectives do the authors bring to this topic?

The authors direct two inclusive postsecondary education (IPSE) initiatives at two universities affiliated with the Pennsylvania Inclusive Higher Education Consortium. These IPSE initiatives provide a full range of academic, social, and residential support to autistic students as they pursue certificates or bachelor’s degrees. Collectively, the authors possess a combined background of over 40 years of experience as either practicing occupational therapists or educators. Their backgrounds supporting individuals with intellectual and developmental disabilities have been honed in state organizations, not-for-profit agencies and clinical settings, and U.S. university systems. The authors also have personal experiences supporting individuals with autism as family members who have observed challenges in educational systems firsthand.

What is already known about this topic?

Previous studies have found that while more autistic individuals are pursuing college, many universities do not have appropriate and coordinated resources or best practices to support these students and their specific needs.

What do the authors recommend?

The authors recommend the following campus-wide initiatives in support of autistic students attending institutions of higher education:

• First, the authors recommend that institutions of higher education implement student-centered planning to hold space for autistic students and identify individualized supports and accommodations needed for a successful college experience.
• Second, the authors recommend that faculty work to foster inclusive practices on campus by engaging in autism and Universal Design Learning (UDL) training so that they can be responsive to students’ accommodations and provide full access to academic learning.
• Lastly, the authors recommend that colleges and universities embed social–emotional learning supports and opportunities for autistic students to build healthy relationships and social capital on their campuses.

How will these recommendations help neurodivergent people now and in the future?

With this *Perspectives Paper*, the authors hope to reach university faculty, staff, and administrators and inspire them to divert necessary funding and resources to foster more coordinated support for autistic students. This enhanced university-level coordination can create a more supportive and inclusive postsecondary experience for autistic students that should lead to greater retention and degree completion of this population.

## Introduction

For many students, obtaining postsecondary education is an exciting opportunity following high school graduation. Autistic students are attending college at higher rates; however, they experience low persistence to graduation due to difficulties in transition from high school to college and a lack of college supports.^1^ In order to promote equal access to postsecondary education, colleges must intentionally increase support and opportunities available to autistic students upon enrollment.

The prevalence of autism in children continues to increase with 1 in 36 children being diagnosed in 2020 compared to 1 in 44 children in 2018.^2^ Data from the 2015 National Autism Indicators Report found that 36% of autistic individuals would attend college during their lifetime.^3^ These data translate to autistic enrollments increasing in U.S. colleges from 1.2% in 2019 to 2.3% in 2023.^4,5^ The true enrollment of autistic students is likely higher as less than half disclose their diagnosis to college personnel; in fact, 75% report rarely disclosing their diagnosis at all.^6^ Research has suggested that autistic students report fearing negative stereotypes associated with disclosing or being uncertain with how or when to disclose their disability.^7–9^ Autistic students can experience bias, micro/macroaggressions, or ableism because members of their campus do not have adequate understanding of their disability. They may also observe a lack of autistic representation in university employees or find these individuals mask as a result of campus culture.

U.S. policy and funding streams (e.g., federal financial aid, Comprehensive Transition Programs [CTP], Medicaid Home and Community-Based Services Waivers, Vocational Rehabilitation) have enabled greater opportunities for autistic students to be able to attend and pay for college.^10–12^ While more autistic students are enrolling in college, the completion rate for these students remains lower than nonautistic peers. Only 38.8% of autistic students persist to degree completion in comparison to 40.7% of peers with disabilities and 52.4% of peers without disabilities.^13^ Data also demonstrate that autistic students delay enrollment in higher education as 47% begin within 6 years of graduating high school, yet only 35% earn degrees in a similar timeframe.^14^ While seminal research by Astin and Tinto^15,16^ note persistence and belonging as key graduation indicators, there is limited scholarship to understand indicators for autistic collegians.

Currently, there are three pathways for autistic students to access higher education in the United States. The first is traditional admissions in degree-seeking programs. The second is through inclusive postsecondary education (IPSE) programs developed through the Higher Education Opportunity Act of 2008 (HEOA) and subsequent Transition and Postsecondary Programs for Students with Intellectual Disabilities (TPSID) grants. IPSE programs support students with disabilities—students with autism spectrum disorders and intellectual and developmental disabilities—to engage in academic coursework, internships, and community activities with nondisabled peers, in pursuit of degrees or nondegree certificates at 343 colleges and universities in the U.S.^17,18^ Although TPSID grants focus on students with intellectual disability, recent data confirm that 26% of TPSID programs host students with dual intellectual disability and autism diagnoses and 6% report being autistic without other diagnosis.^19^ A third pathway to higher education is through Autism-Specific College Support Programs (ASPs), focusing specifically on degree-seeking autistic students. While there were 74 programs situated at 2- and 4-year institutions during the 2017–2018 academic year, 21 states still did not offer any level of autism-focused IPSE or ASP programming in 2021.^20^ Public institutions are more than twice as likely to host ASPs than their private counterparts.

Regardless of how autistic students are accessing higher education, universities have a responsibility to support the diverse needs of these students to improve retention and graduation rates. We write from the vantage point of overseeing U.S. IPSE programs that support students with intellectual and developmental disabilities, with several of our students identifying as autistic, and issue a call to action to our colleagues in higher education to reconsider how we support autistic students. While not all universities have IPSE/ASP options, universities can utilize preexisting infrastructure and intentional collaboration among departments to support autistic students.^21^

## Summary of Current Literature

This *Perspectives Paper* summarizes literature from the last 10 years on autistic students’ postsecondary experiences and university best practices in the United States. With the passage of the HEOA in 2008,^22^ development of the College Autism Network in 2014,^23^ and an increase in the number of IPSE/ASP programs over the last decade, a growing body of literature has begun to shed light on the postsecondary experiences and unmet support needs of autistic college students. While many scoping and systematic reviews referenced below include international studies, the focus of this paper is on autistic students in U.S. postsecondary settings. Therefore, the literature summarized below highlights themes applicable to the United States, which may not generalize to international contexts.

Recent systematic and scoping reviews on the autistic student experience note significant challenges experienced by autistic students during transition to college, inconsistencies in the level of support provided on university campuses, and suggestions that may help close social and educational disparity gaps for them.^24–33^ However, there is a dearth of evidence-based literature on tangible outcomes associated with the best practices proposed in these articles.

### Skill development

Several reviews focused on the skills autistic postsecondary students (or parents, caregivers, or support staff) identified as necessary for successful transition to college.^28,30,33^ Studies conducted by Davidson et al.,^34^ McLeod et al.,^35^ and Wischnewsky^33^ all found that autistic students have difficulty developing executive functioning, self-regulation, self-determination, and self-advocacy skills during transition to and during college. It is imperative to build a solid foundation of these skills prior to going to college, ideally in high school or summer transition programs, since autistic students’ executive functioning, emotional, and behavioral needs are not protected or supported by the Americans with Disabilities Act or Section 504 of the Rehabilitation Act once they get to college.^33,36^ A lack of support in developing these skills can affect how students engage socially, manage their time, and complete academic and nonacademic tasks.

### University best practices

White et al.^37^ assert that necessary first steps to universities supporting and embracing neurodivergent students are institutional support and changing campus attitudes toward autistic students. While this change can be gradual, the quality of interactions between neurotypical and neurodivergent faculty, staff, and students is one of the largest predictors of campus-wide attitudinal change and can decrease neurodivergent students’ fear of stigmatization, especially related to disclosure of their diagnosis.^37,38^

Autistic representation on university-level decision-making organizations (e.g., Faculty Senate, student organizations) can also lead to positive interactions between neurotypical and neurodivergent campus stakeholders, and the needs of autistic students considered and infused into university systems.^39^ Training the campus community on autism and neurodivergent student needs has had variable effects on stakeholder knowledge and attitudes toward this student population; however, one element that’s widely recognized as effective is involving neurodivergent students/faculty/staff as codevelopers and facilitators of trainings.^39–41^ More research is needed on influencers of attitudinal change toward autistic students in university settings.

Two recent scoping reviews highlighted the best practices for supporting autistic students in postsecondary settings in the United States, United Kingdom, Australia, and Canada, with the majority of articles coming from the United States.^31,32^ Academic accommodations were most frequently cited as a valuable campus support followed by social and emotional support. Supports in the following areas were deemed highly valuable to neurodivergent students; however, they were rarely researched and represent a potential gap in university supports: sensory accommodations, communication/self-advocacy, employment, financial management, service navigation, relationship development, and sexual health.

### Mentorship and coaching

Peer mentor and faculty relationships are identified in the literature as critical nonacademic and academic supports for autistic students in postsecondary settings.^26,33,42,43^ Peer mentors are neurotypical or neurodivergent students matched with an autistic peer on age, academic major, class year, or shared interests/experiences. They provide support in the areas of academic tutoring, functional life skills (e.g., self-advocacy, self-determination, organization, and time management), and social–emotional learning to enhance inclusion on-campus.^31,44,45^ Rando et al.^45^ likened peer mentors to “transition coaches” who provide support to autistic peers as they navigate a new college environment. Mentors and mentees meet weekly or biweekly for 1–10 hours a week depending on a student’s individualized needs.^31^ Mentoring supports are typically organized through IPSE/ASP programs and, in rarer cases, via university Disability Services offices.^28^

Many studies included in Nelson et al.’s^32^ scoping review on autistic postsecondary supports found that through structured peer mentorship, autistic students’ social–emotional learning improved through the formation of genuine, reciprocal interpersonal connections. Of the nine studies in Duerksen et al.’s^26^ review, five found that autistic students were more engaged socially and felt a greater sense of belonging on campus as a result of peer mentoring, and three detailed positive academic outcomes including improved GPA, self-efficacy, self-regulation, time management, and organizational strategies. Rando et al.^45^ also found that first-year retention rates increased for autistic students and faculty concerns over support of these students decreased in response to a year-long peer mentorship program. While these findings are promising, most mentorship research is limited in scope by small sample sizes and few studies examine the long-term benefits of mentoring on lifelong reciprocal friendships, social capital, and mental health outcomes for autistic students.^26,46^

Faculty are an equally important source of support for autistic students as they facilitate student access and engagement in academic courses, provide career mentorship, and gauge a student’s adjustment to college. Faculty attitudes toward inclusion and commitment to fostering inclusion are established predictors of successful inclusive education initiatives.^17,47^ Research also suggests that underrepresented and minority students who have positive faculty mentors are more likely to graduate.^48,49^ Across multiple studies, however, faculty expressed a hesitancy and lack of preparedness to support the unique needs of autistic students.^43,50^

While little research has examined faculty mentoring of autistic students, universal design for learning (UDL) or universal design for instruction (UDI) has been identified as an effective in-class support for students with disabilities.^43,51,52^ UDL is a framework that encourages faculty and students to engage in multiple, varied methods of participation and expression (i.e., visual, auditory, kinesthetic, and narrative) throughout a course to promote learning that is more inclusive and accessible. UDL aims to create an equitable educational playing field for students with disabilities and has demonstrated benefits for all students^53^; however, as high as 55% of university faculty reported a lack of understanding of how to utilize UDL in their classrooms and course design.^54–56^

Current literature on autism and postsecondary settings is largely focused on the individual skills and services (e.g., Disability Services, Peer Mentors) that autistic students need to be successful in college, while little attention is paid to a university’s responsibility to support a student’s persistence to graduation, nor their quality of life on campus. Institutions of higher education may argue that by offering general campus/disability services, they have fulfilled their obligation to students; however, as profiles of collegians become more complex (i.e., multiple intersectional and minoritized identities), institutions must expand and adapt to changing populations. Furthermore, universities must work to reduce campus silos by aligning academic and cocurricular supports to ensure students receive resources they require. To date, few articles propose a coordinated effort on behalf of universities to better support autistic students in postsecondary education.^57,58^

## A Call to Action

It is our position that campuses must intentionally implement coordinated efforts to support autistic students. The idea of a specific campus unit supporting underrepresented students is not novel^39^ as many universities already offer support for students who identify as veterans, first generation, international, members of the LGTBQIA+ community, and a person of color or with a disability; however, these intentional supports are often lacking for autistic students.^59^

We recommend that, if not already established, a campus unit be created to support autistic students by leveraging and coordinating with existing campus resources (e.g., disability services, academic advisement, counseling, residence life) to reduce administrative barriers that arise during autistic students’ college experiences. Additionally, as autistic students transition to college, there must be greater transparency in distinctions between K-12 and higher educational systems so that students and their families understand what resources are available.^33^ Colleges must educate prospective students not only on the difference in accommodations available to them but also on the rigorous level of documentation required to receive accommodative support in college.

Our team asserts that it is imperative campuses create systems that infuse neurodiversity-affirming approaches, and strategies that promote acceptance of neurodivergence as opposed to valuing neurotypicality, in support of an autistic student’s higher education experience.^60,61^ This article proposes that coordinated efforts at the university level become the best practice for autistic students in higher education in order to promote authentic and inclusive college experiences for this growing student population. Table 1 presents the on-campus units we recommend autistic students and IPSE/ASP programs interface with regularly to enhance students’ experiences, and the facilitators and barriers students may experience as they navigate these campus supports.

**Table 1.** Facilitators and Barriers to Accessing University Supports for Autistic Students

University office	Facilitator(s)	Barrier(s)
Disability/accessibility services	● Empower students to disclose their disability and increase campus autism awareness^62^ ● Act as a liaison to campus units who support accessibility ● Sponsor campus disability advocacy group or create opportunities to meet other neurodivergent students^62,63^ ● Autistic students attribute successful college completion to positive relationships with Disability Services staff^64^	● Units may have high caseloads causing access challenges for autistic students^62^ ● Offices may not have capacity to offer additional resources (e.g., academic coaching, peer mentor support) for autistic students ● Staff may lack training to support autistic students^62^ ● Accommodations are often standardized and lack individualized supports^62,65^
Title IX/student conduct	● May develop plain language educational guides and student policies/handbooks ● Preventatively support students in decision-making and social interaction cues^66^ ● Encourage restorative justice processes for students who may have campus incidents^67^ ● May implement/coimplement Elevatus Sexuality Educator training for the campus^68^	● Many universities still do not offer plain language policies (e.g., student handbooks, Codes of Conduct, Title IX and other administrative policies) ● Title IX and Student Conduct proceedings may not consider communication and/or neurotype differences during interviews, hearings and deliberation processes ● Commonly used Title IX trainings may use content that is difficult for autistic students to understand (e.g., the Tea Consent video)^69^
Career services/development	● National Association of Colleges and Employers and National Career Development Association standards mandate career counselors to serve all student users^70,71^ ● Support autistic students in seeking information about jobs, internships and direct communication with employers^72^ ● Focused career sessions for autistic students on interview/soft skill development, career matching based on their strengths and interests, and barriers to career search^73^ ● Encourage autistic students to attend on- and off-campus career fairs and use the Handshake platform and “The Big Interview” resources^74,75^	● Autistic students may not actively seek out career services for help with securing employment opportunities^72,76^ ● Autistic students may require alternative career evaluations due to the way they process information^73^ ● Career counselors must tailor their support for autistic students in light of research that demonstrates that autistic individuals are frequently hired into entry-level jobs lower than their credentials, receive lower wages and have difficulty maintaining jobs^73,77–85^
Campus police	● Serve as resources for autistic students when addressing crisis or other safety concerns ● Connect autistic students to campus educational programming and training opportunities (e.g., safety, alcohol and other drugs, first aid/CPR, NARCAN, Stop the Bleed, and self-defense)	● Campus police officers may not be formally trained in autism-specific intervention and de-escalation strategies^86–88^ ● Campus police units may still use punitive versus developmental models when engaging students
Student involvement and campus activities/recreation and intramurals	● Campus involvement predicts student retention and degree completion for all undergraduate students^15,16^ ● Autistic students’ transition to college are positively or negatively impacted by social relationships developed on campus^89^ ● Autistic students positively identify connecting to peers with similar interests through clubs and campus activities^90,91^ ● Recreational activities are pathways to inclusion for people with disabilities^92^	● College students with IDD (including students with ASD) have not been involved in all forms of campus recreation^92^ ● Autistic students may have visual, auditory or olfactory stimuli triggers that are not often considered in the planning of campus programming and activities^93,94^ ● Accessibility for club sports, recreation and intramural programs can prove problematic for autistic students who have sensory concerns^92^ ● Peer mentors may not be utilized by Student Involvement units ● Recreation staff often lack skills and training to include individuals with varying abilities^92,95^
Residence life/university housing	● Campus housing professionals assist in holistic development of students with and without disabilities at a deeper level than other campus units^96^ ● On-campus housing offers opportunities for personal skill development in independence, social engagement, and activities of daily life^97^ ● On-campus housing experiences connect students to peers, academic success, personal growth and degree persistence^97–101^ ● Autistic students can serve as collaborators (e.g., Resident Assistants) in housing models as well as with programming^97,102^	● Student satisfaction can be impacted by housing environment^103^ ● On-campus housing was designed without consideration of neurotype and may cause stress or other challenges for autistic or other marginalized students^97,104–106^ ● Campus housing staff often lack knowledge (or training) about neurodiversity, inclusive language, accommodations, or legal requirements^107,108^ ● Autistic students may become overwhelmed around peers, but find living without roommates isolating if they choose a single housing arrangement^28,109,110^
Financial aid	● Financial Aid staff can assist autistic students in accessing available funding sources to help pay for college (e.g., federal financial aid, Autism Waiver or Home and Community-Based Services Waiver funds, Office of Vocational Rehabilitation funds, 529 plans)	● Differing enrollment statuses (e.g., full-time, part-time, credit vs. noncredit bearing) can impede access to Waiver funds, federal financial aid, etc. ● Financial Aid staff must be aware of the multitude of funding sources that autistic students are eligible for ● University Satisfactory Academic Progress (SAP) policies may not consider neurotype difference in assignment of specific GPA/QPA requirements

We highlight the following three recommendations based on literature that establishes faculty, staff and peer attitudes and relationships as positive predictors of student inclusion, belonging and retention on college campuses and our personal experiences as IPSE directors.^15,16,37,38^ While individualized accommodations are expressed as a desired support among autistic students in the literature,^32^ they are still emerging as a best practice area in IPSE settings; however, we have seen early success brokering these accommodations with Disability/Accessibility services and faculty on our respective campuses. Our recommendations are detailed below and summarized in Table 2.

**Table 2.** Summary of Author Recommendations

Recommendations
Recommendation 1—Establish person-centered planning that includes individualized accommodations ● Support students in person-centered planning and using goal-setting tools while at college ● Shift toward individualized accommodations driven by student need
Recommendation 2—Engage faculty as partners in inclusive best practices ● Provide faculty with autism awareness and Universal Design for Learning (UDL) training ● Encourage faculty to incorporate UDL principles within the classroom ● Establish faculty mentors for autistic students
Recommendation 3—Embed opportunities and support for social–emotional learning and relationship building ● Increase accessibility of campus events ● Establish self-advocacy groups ● Utilize peer mentors who are matched to an autistic student’s interests and needs

## Recommendation 1: Establish Person-Centered Planning that Includes Individualized Accommodations

Colleges must actively implement person-centered planning (PCP) for autistic students to ensure that a wide range of their needs are met. Rising collegians (admitted students who have not yet begun college) with IEPs should already be familiar with these plans as some variation of them is used during Individuals with Disabilities Education Act (IDEA)-required transition planning.^111,112^ Unfortunately, many college students may struggle to demonstrate autonomy (i.e., ownership) over their educational outcomes in postsecondary education as they relied heavily on teachers and/or family members in previous settings.^112–115^ Through the incorporation of PCP, autistic students will be supported in advocating for individualized accommodations, accessing campus supports, and identifying experiences related to their college and career goals.

### PCP and goal setting

Recent college admissions processes have shifted toward student-centric artifacts (e.g., video prompts and essays) to identify prospective students’ compatibility with academic programs, institutional fit, and campus culture. These artifacts can also inform campus stakeholders of potential needs of incoming students and trigger referrals to campus resources. In similar ways, PCP is a process regularly used with individuals with disabilities and their support networks to identify their goals in areas such as education, healthcare, employment, housing, relationship, and skill building.^116^ Formal PCP and goal setting tools such as Planning Alternative Tomorrows with Hope, Charting the LifeCourse, or the Foundational Skills for College and Career Learning Plan^117–119^ give students the central voice in the process and enable them to express their strengths, interests, goals, and support needs to inform accommodations more effectively than IEPs or other academic documentation. Well-executed plans can be titrated down to one-page profiles which a student can use in conversations with faculty and staff who support them on campus.

### Individualized accommodations

PCP is far preferable to the generalized accommodations regularly offered to students with disabilities such as extended time on examinations/assignments, noise-free testing environments, or advanced provision of faculty notes/lectures.^28,120,121^ Universities must shift toward individualized accommodations driven by the needs of the student as these impact autistic students in areas such as time management, study skills, social anxiety, sensory sensitivity, and executive function.^26,28,57^ A campus will not authentically support neurodivergent students if there is not implicit recognition that autism is a natural neurological variant, and students should not be pathologized.^120^

There should be no reason why an institution is unable to fulfill accommodation requests, presuming they are not financially prohibitive or deemed unreasonable or extraordinary. This is achieved by first recognizing that accommodations that involve cognitive, sensory, social, and regulatory domains^122^ can be developed and remain in compliance with legal standards. Moving a classroom location, for example, to accommodate sensorial needs (e.g., lighting, climate control) illustrates a reasonable accommodation. Requesting alternative housing with kitchen options on campus’ where these may not already exist, to avoid sensory issues in a dining hall may conversely be deemed unreasonable.^121^ Universities should proactively assist students to identify alternative supports that exist beyond campus (e.g., provider agency support, Waiver funds, Vocational Rehabilitation resources, etc.) if/when an accommodation is declined.

In the years we have overseen programs, we cannot underscore the importance of autistic students advocating for individualized accommodations which not only support their academic success but can be utilized later for success in the workforce, community, or personal lives. It is noteworthy that, while scholarship on autistic students and academic accommodations has increased over the past decade, literature on accommodating students in nonacademic domains remains limited.

## Recommendation 2: Engage Faculty as Partners in Inclusive Best Practices by Providing Autism and UDL Training

With significant increases in the number of autistic students pursuing college degrees,^123,124^ faculty are key partners in ensuring students feel welcome, included and engaged in academic courses of study. Faculty willingness to teach and mentor autistic students is directly related to their understanding of autism and prior experiences with autistic people.^43,125^

### Faculty training

Fostering faculty buy-in as partners in inclusion is vital to the success of inclusivity at a university. This can occur through faculty training in autism, PCP, and UDL/UDI principles, and can be coordinated by university Centers for Teaching and Learning via asynchronous or synchronous online courses, or in-person workshops. In Waisman et al.’s^126^ study, faculty who engaged in only two asynchronous training modules (codesigned by autistic and neurotypical people) on autism and UDL held significantly lower stigma toward autistic students; 87% of these faculty reported using UDL in their teaching. Faculty who maintain professional licensure (e.g., education, special education, health sciences) and/or are seeking tenure and promotion are further incentivized to seek continuing education units and microcredentials in UDL/UDI and infuse it into their teaching practices.

### Incorporating universal design for learning

Centers for Teaching and Learning offer one-to-one support for faculty in course design, assignment and rubric development, and innovative teaching strategies. IPSE/ASP program staff and Offices of Disability Services can work with autistic students and faculty to identify course/assignment adaptations in line with reasonable accommodations. Faculty can infuse UDL/UDI creatively by providing daily schedules of class activities; giving students flexibility to choose individual versus group work and different assignment formats, (e.g., video/presentation in place of writing assignments); incorporating photographs/video rather than textual information; and using a scaffolding approach to course content and assignments or flipped classrooms.^53,125,127^ For example, faculty affiliated with our IPSE programs have opted for a “Choose Your Own Adventure” learning format in their courses. This allows students to choose a variety of methods that are best suited to them to demonstrate their learning; the only caveat is that to earn a passing grade, students must choose a combination of assignments to reach a predetermined point threshold. Faculty who regularly use UDL techniques note increased learning outcomes for students with and without disabilities; this is more important as universities grapple with low student engagement and academic performance following the COVID-19 pandemic.^128–131^ Faculty on our campuses who have used a UDL/UDI lens to redesign their courses report that this process has made them better teachers. Neurotypical students also expressed that they valued having autistic students in their courses, and this daily interaction fostered greater openness toward inclusion.^17^

### Establish faculty mentors

Faculty can play a pivotal role in recruiting autistic students to university campuses and providing advice and mentorship once they arrive. In Accardo et al.’s^132^ study on desired campus supports and services, 91% of autistic students requested academic coaching and 48% expressed the desire to have an experienced faculty mentor. Faculty mentors can foster skill building in academic/study skills, planning and organization, time management, professional skills, and career development (e.g., resume development, networking with future internships/employers).^133^ Mentoring relationships between faculty and autistic students can occur organically based on individual interests. However, many of the IPSE programs affiliated with Pennsylvania’s Inclusive Higher Education Consortium^134^ have implemented an intentional faculty/staff advisement model (in addition to students’ academic advisors) in which faculty or staff are paired with autistic mentee(s). These faculty–student pairs meet weekly so students can share their successes, express their needs, and link with appropriate on-campus resources, as needed.

## Recommendation 3: Campuses Should Embed Opportunities and Support for Social–Emotional Learning and Relationship Building

Attending college provides all students with an opportunity to further develop their social–emotional capabilities and fill valued social roles such as friend, classmate, partner, roommate, and coworker. Autistic students often feel unprepared to meet these social expectations as they transition into the complex context of a university.^135^ In addition, most autistic students experience significant social and emotional challenges when transitioning to college, have lower quality relationships on campus compared to nonautistic peers, and report that, despite receiving academic accommodations, they lacked social–emotional and functional life skills support.^35,136,137^ Universities must incorporate supports and opportunities for autistic students to develop similarity and regularity with peers by creating opportunities such as common hours, informal gatherings or structured “meetups.”^138^

### Increase accessibility of campus events

Universities offer numerous social opportunities, including clubs, student organizations, and campus events, that allow students to form meaningful relationships and a sense of belonging within the campus community. For autistic students, many of these events are not accessible due to the overstimulating sensory environment;^125^ having sensory friendly spaces and events is desired by many autistic students.^121^ Universities must analyze sensory environments and accessibility of their campus spaces. Making relatively small changes such as increasing natural lighting, providing sensory aids (i.e., fidgets and headphones), allocating quiet spaces, and providing visual signage can significantly increase a student’s ability to participate in events.^139^ Sensory-friendly spaces should be designed in residence halls, libraries, student unions, recreation centers, dining facilities, and programming spaces. Additionally, universities should consult autistic students/staff when designing and implementing these changes to meet the needs of this population. Having equal access to campus events and spaces will provide autistic students with increased opportunities to develop social connections with their peers.

### Establish self-advocacy groups

Neurodiverse self-advocacy groups provide autistic students with the opportunity to connect with peers in a welcoming environment; however, these groups are not present on many university campuses. We recommend that campuses support autistic-led advocacy groups by encouraging students to come together on a regular basis. In this semistructured setting, students can meet other neurodivergent peers, find common interests, share strategies for success at college, and collaboratively advocate for increased accessibility on campus. Members of self-advocacy groups can increase their social capital while connecting with peers living a similar college experience.

### Utilize peer mentors

Peer mentorship is another support that can be used to enhance relationship building for autistic students. Peer mentors provide direct instruction and guidance and model a variety of social–emotional skills within the natural context of the university. We recommend that peer mentorship occur within preexisting academic/social events or campus groups to promote full inclusion of autistic students. This may take the form of a student and their peer mentor attending a club together, during which the mentor would support the student to navigate the social landscape of a college organization. This model of peer mentorship increases autistic students’ social engagement and participation in social events on campus.^31,140^ In addition, several mentor–mentee relationships within our programs have evolved into reciprocal friendships that last beyond the students’ time at college. To improve the mentorship model, universities should begin to employ more autistic peer mentors. Despite a gap in the literature, based on our experience employing neurodivergent peer mentors, these mentors can draw upon their own experiences with accessing supports and building relationships within the university structure to support their mentee and create a safe environment for social–emotional learning.

## Conclusion

The rise in autistic students seeking postsecondary education is positive on multiple levels. Students obtain higher education credentials in line with their interests, which promotes self-efficacy, self-determination, personal fulfillment, and closes educational, employment, and socioeconomic gaps for this population. Neurotypical peers and university faculty, staff and administration reciprocally benefit by expanding their understanding of autism and openness to inclusion in higher education spaces. Faculty and staff who embrace UDL in response to neurodivergent students improve outcomes for all students and become more creative and responsive professionals in the process. Siloed university systems that are not prepared to support the unique needs of autistic students can do these students harm rather than bolstering them educationally, emotionally, and socially.

This paper is a call to universities to design more coordinated systems of support for autistic students by engaging multiple campus stakeholders and resources. Universities must also expand future research efforts to monitor: (a) census data and the rate of autistic students seeking postsecondary education; (b) campus attitudes (and predictors of attitudes) toward neurodivergent students; and (3) outcomes of neurodiversity-affirming initiatives (e.g., peer/faculty mentoring and UDL) to bolster advocacy efforts with university administration and outside funders. Coordinated university efforts individualized to autistic students’ needs can serve as the foundation for a positive college experience and, in turn, a meaningful and fulfilling life.
